# Antecedent Disease and Amyotrophic Lateral Sclerosis: What Is Protecting Whom?

**DOI:** 10.3389/fneur.2016.00047

**Published:** 2016-03-29

**Authors:** Sabrina K. Hollinger, Ike S. Okosun, Cassie S. Mitchell

**Affiliations:** ^1^Department of Biomedical Engineering, Georgia Institute of Technology, Emory University, Atlanta, GA, USA; ^2^Epidemiology and Biostatistics, School of Public Health, Georgia State University, Atlanta, GA, USA

**Keywords:** motor neuron disease, premobidity, comorbidity, preexisting condition, epidemiology

## Abstract

Multiple studies have shown that antecedent diseases are less prevalent in amyotrophic lateral sclerosis (ALS) patients than the general age-matched population, which suggests possible neuroprotection. Antecedent disease could be protective against ALS or, conversely, the asymptomatic early physiological underpinnings of ALS could be protective against other antecedent disease. Elucidating the impact of antecedent disease on ALS is critical for assessing diagnostic risk factors, prognostic outcomes, and intervention timing. The objective of this study was to examine the relationship between antecedent conditions and ALS onset age and disease duration (i.e. survival). Medical history surveys for 1439 Emory ALS Clinic patients (Atlanta, GA, USA) were assessed for antecedent hypertension, hyperlipidemia, diabetes, obesity, asthma, arthritis, chronic obstructive pulmonary disease (COPD), thyroid, kidney, liver, and other non-ALS neurological diseases. The ALS onset age and disease duration are compared between the antecedent and non-antecedent populations using chi square, Kaplan–Meier, and ordinal logistic regression. When controlled for confounders, antecedent hypertension (high blood pressure), hyperlipidemia (high cholesterol), arthritis, COPD, thyroid disease, and non-ALS neurological disease are found to be statistically associated with a delayed ALS onset age, whereas antecedent obesity [body mass index (BMI) > 30] was correlated to earlier ALS onset age. With the potential exceptions of liver disease and diabetes (the latter without other common comorbid conditions), antecedent disease is associated with overall shorter ALS disease duration. The unique potential relationship between antecedent liver disease and longer ALS disease duration warrants further investigation, especially given liver disease was found to be a factor of 4–7 times less prevalent in ALS. Notably, most conditions associated with delayed ALS onset are also associated with shorter disease duration. Pathological homeostatic instability exacerbated by hypervigilant regulation (over-zealous homeostatic regulation due to too high regulatory feedback gains) is a viable hypothesis for explaining the early-life protection against antecedent disease and the overall lower antecedent disease prevalence in ALS patients; the later ALS onset age in patients with antecedent disease; and the inverse relationship between ALS onset age and disease duration.

## Introduction

Amyotrophic lateral sclerosis (ALS) is a debilitating neurodegenerative disease with a relatively unknown etiology. Only 5–10% of the ALS population can be attributed to familial factors (1), while the rest are sporadic. Existing research has found several factors that impact disease duration and age of onset of ALS in patients. Onset age has been found to occur earlier in those of Asian race or male gender (2, 3). Shorter disease duration is related to patients who have a later onset age of ALS symptoms (2, 4–6).

Many times, those diagnosed with ALS have previously led healthy and active lifestyles (7, 8). In fact, it has been repeatedly shown that ALS patients have lower rates of antecedent disease than that of the general public (9–11). Our prior foundational study comprehensively assessing the prevalence of antecedent disease found that arthritis, non-ALS neurological disease, liver disease, chronic obstructive pulmonary disease (COPD), kidney disease, asthma, diabetes, hypertension, obesity, and hyperlipidemia were all statistically less prevalent in ALS compared to age-, gender-, and geography-matched control patients (11). Thyroid disease was qualitatively less but statistically similar to the matched control population (11). Lower overall prevalence of antecedent disease in ALS populations suggests possible hypotheses of neuroprotection. That is, antecedent disease could be protective against ALS, or conversely, the early or asymptomatic pathophysiological underpinnings of ALS could be protective against other antecedent diseases (11).

While there is a general consensus that antecedent disease is less prevalent in ALS, the actual impact of antecedent disease on ALS disease duration is more controversial (9, 12–15). Smaller studies examining the relationship of antecedent diseases to ALS survival have had varied results, including positive (15–17), neutral (9, 13), or negative (5) effects on survival duration of ALS in patients. Interestingly, the results of studies assessing the relationship between antecedent disease and ALS onset age have been more consistent, finding that ALS patients with antecedent disease generally have a later onset age (9, 12). Here, we perform the first large-scale (*n* = 1439 ALS patients) study to examine if multiple types of antecedent conditions affect ALS diagnosis age and disease duration.

## Materials and Methods

A case–control study of antecedent conditions on ALS onset or diagnosis age and disease duration of an ALS population was performed. The ALS population consisted of 1439 patients from the Emory ALS Clinic, in Atlanta, GA, USA, confirmed to have ALS at their first clinic visit by a board-certified neurologist *via* the use of exclusionary diagnostic criteria (MRI, blood tests, electrophysiology, CSF, etc.) and/or in-clinic physical exam. The Internal Review Boards of Emory University, Georgia Institute of Technology, and Georgia State University approved this study. Due to the nature of this retrospective study, patient consent was waived by the Internal Review Boards.

### Antecedent Conditions

The antecedent presence of hypertension (high blood pressure), hyperlipidemia (high blood serum cholesterol, also called dyslipidemia), diabetes, obesity, arthritis, asthma, COPD, thyroid disease, neurological disease (excluding ALS), kidney disease, and liver disease were assessed at the first ALS clinic visit by trained medical personnel using a standardized comprehensive medical history patient entry survey. Additional information for gathering the data can be seen in our previously published work, which laid the foundation for the current study (11, 18).

### Study Populations

A total of 1439 confirmed ALS patients were included in the study. The control population consisted of 600 confirmed ALS patients who had none of the assessed antecedent conditions at ALS onset. The antecedent conditions population consisted of 839 confirmed ALS patients who had at least one antecedent condition at the ALS onset. Given that all patients had a recorded ALS onset age, all 1439 patients were included in the ALS onset age study group (600 control patients and 839 antecedent disease patients). Of the 1439 patients in the ALS onset age study group, 787 patients had a recorded date of death and were additionally included in the ALS disease duration study group (354 control patients and 433 antecedent disease patients).

### Statistical Analysis

All statistical tests were completed using SAS 9.3. Chi square analysis was done to compare the distributions of the control population and antecedent condition population for both outcomes of interest. For the age of onset and disease duration, the chi square compared the distribution above and below the mean. Additional chi square analysis was done looking at the extremities of the population. This method was utilized to assess the potential impact of patient age on the findings. That is, we assessed whether younger or older ALS patients have a different distribution. For the age of onset, the chi square compared the distribution above and below the middle standard deviation (SD) of onset age of the entire population or those who were either older than 66.4 years or younger than 53.9 years. Chi square analysis compared the disease duration distribution above and below the middle SD of disease duration in a subset of the total population or outside the range of 1.1–3.2 years.

Kaplan–Meier curves showed the disease duration for a qualitative assessment. Specifically, the Kaplan–Meir curves plot the survival probability versus disease duration (in years) for the antecedent and control subgroups within the ALS disease duration study population. All individual antecedent conditions, along with groupings of conditions, were analyzed.

Ordinal logistic regression was completed for both age of onset and disease duration to assess for confounders and compare the antecedent conditions with known risk factors. This was done to examine what extent each of the antecedent conditions and other factors related to the age of onset and disease duration.

For both models, male gender and Caucasian race were set as the reference group. Race was assessed as Caucasian, African-American, and other. The ordinal logistic regression for age of onset included all antecedent conditions, race, and gender. The ordinal logistic regression for disease duration included all antecedent conditions, race, gender, and age of onset.

## Results

We present the demographics and statistical assessment of the association of antecedent disease with ALS onset age or disease duration in a population of 1439 ALS patients using chi square, Kaplan–Meier, and ordinal logistic regression.

### Demographics

The ALS onset age study group (consisting of those who had a confirmed ALS diagnosis and recorded ALS onset age) has 60% males and 40% females, with 58% of the population having at least one antecedent condition. The ALS disease duration study group (consisting of those with a confirmed ALS diagnosis, recorded onset age, and recorded date of death) has 57.7% males and 42.3% females, along with 55% having at least one antecedent condition. Gender falls on a more equal distribution the disease duration group compared to the onset age group (*p* < 0.05). All of the antecedent conditions except hyperlipidemia, diabetes, and obesity occur within the same distribution between both groups (see Table 1 for additional demographic information).

**Table 1:** **Demographics of Emory Clinic ALS population by ALS age of onset and disease duration**.

Population characteristics	Onset age group			Disease duration group^a^		
	Overall	Control	Disease	Overall	Control	Disease
*N*	1439	600	839	787	354	433
Age^b^^,^^d^						
Mean (SD)	60.1 (12.5)	56.0 (13.4)	63.1 (11.0)	61.2 (12.4)	57.1 (13.3)	64.6 (10.5)
Disease duration (years)						
Mean (SD)^d^	–	–	–	2.1 (2.1)	2.4 (2.3)	1.9 (1.9)
Gender^c^% (*N*)						
Male^d^	60.0 (864)	63.2 (379)	57.8 (485)	57.7 (454)	62.7 (222)	53.6 (232)
Female^d^	40.0 (575)	36.8 (221)	42.2 (354)	42.3 (333)	37.3 (132)	46.4 (201)
Race% (*N*)						
Caucasian	57.4 (826)	56.5 (339)	58.3 (489)	59.1 (465)	56.5 (200)	61.2 (265)
African-American	12.3 (177)	12.3 (74)	12.3 (103)	10.7 (84)	10.7 (38)	10.6 (46)
Other	30.2 (434)	31.2 (187)	29.6 (247)	30.2 (238)	32.8 (116)	28.2 (122)
Antecedent conditions% (*N*)						
Hypertension	36.9 (531)	–	63.3 (531)	35.8 (282)	–	65.1 (282)
Hyperlipidemia^c^	26.3 (378)	–	45.1 (378)	24.0 (189)	–	43.6 (189)
Diabetes^c^	9.0 (129)	–	15.4 (129)	7.9 (62)	–	14.3 (62)
Obesity^c^	9.1 (131)	–	15.6 (131)	7.2 (57)	–	13.2 (57)
Arthritis	5.1 (74)	–	8.8 (74)	5.0 (39)	–	9.0 (39)
Asthma	4.9 (71)	–	8.5 (71)	4.8 (38)	–	8.8 (38)
COPD	3.1 (45)	–	5.4 (45)	2.8 (22)	–	5.1 (22)
Thyroid	6.2 (90)	–	10.7 (90)	6.1 (48)	–	11.1 (48)
Neurological disease	0.8 (11)	–	1.3 (11)	1.0 (8)	–	1.8 (8)
Kidney disease	0.8 (12)	–	1.4 (12)	1.3 (10)	–	2.3 (10)
Liver disease	0.8 (12)	–	1.4 (12)	0.9 (7)	–	1.6 (7)

*The overall study population consisted of 1439 ALS patients (600 control patients and 839 antecedent disease patients). All ALS patients were included in the onset age group. The subset of patients in the ALS onset age that had a recorded date of death was also included in the disease duration group*.

*^a^Disease duration population is a subgroup of diagnosis age population*.

*^b^Age at first visit to clinic*.

*^c^Age of onset population significantly different than disease duration population (*p* < 0.05)*.

*^d^Control population significantly different than antecedent condition population (*p* < 0.05)*.

The ALS onset age study group has a diagnosis age mean of 60.1 years with a SD of 12.5 years. The mean diagnosis age among the control population was 56.0 years, males at 54.4 years, and females at 58.9 years of age. Those with antecedent conditions had an average of 63.1 years, males at 62.4 years, and females at 64.1 years of age. As has been previously shown, a substantially greater proportion of individuals with antecedent conditions are older when compared to those without antecedent conditions of interest (*p* < 0.0001).

The ALS disease duration study group has an average disease duration of 2.1 years with a SD of 2.1 years. Those without antecedent conditions of interest have an average duration of 2.4 years with males at 2.5 years and females at 2.3 years, respectively. Those with antecedent conditions have disease duration of 1.9 years with males at 2.0 years and females at 1.8 years, respectively. The antecedent condition group had a substantially shorter disease duration when compared with those without antecedent conditions (*p* < 0.0001).

### Chi Square Analysis

Chi square test was used to compare age of onset and disease duration between antecedent conditions and those without any antecedent conditions. Antecedent condition populations include sole condition (only having one antecedent condition of interest), multiple condition (having multiple antecedent conditions, including the condition of interest), cardiovascular (having any number of the following: hypertension, hyperlipidemia, diabetes, or obesity), autoimmune (having any of the following: asthma, arthritis, COPD, thyroid disease), and amount (the number of antecedent conditions present). For example, a patient with the conditions of diabetes and asthma would be located in the multiple condition group for asthma and for diabetes, the cardiovascular group, the autoimmune group, and the amount group of 2.

Sole and multiple condition populations are found in Table 2. Cardiovascular, autoimmune, and amount populations are found in Table 3. Mean age of onset and disease duration of the antecedent condition populations are reported along with the number of patients analyzed for that comparison within the tables.

**Table 2:** **The effect of individual antecedent conditions on ALS age of onset and disease duration determined from chi square analysis**.

Population	Age of onset			Disease duration		
	Mean (SD)	*N*^b^	*p*-value	Mean (SD)	*N*^b^	*p*-value
**Control**^**^a^**^	56.0 (13.4)	1439	<**0.0001**	2.4 (2.3)	787	<**0.0001**
**Antecedent condition**						
*Hypertension*						
Sole	63.2 (10.5)	787	<**0.0001**	1.9 (1.9)	461	**0.0129**
Multiple	64.3 (10.5)	1131	<**0.0001**	1.8 (1.7)	636	<**0.0001**
*Hyperlipidemia*						
Sole	62.4 (10.0)	687	<**0.0001**	2.0 (1.5)	390	0.1700
Multiple	65.3 (10.2)	978	<**0.0001**	1.7 (1.5)	543	<**0.0001**
*Diabetes*						
Sole^c^	50.9 (14.4)	612	0.1439	3.3 (3.0)	362	1
Multiple	63.9 (10.1)	729	<**0.0001**	1.7 (1.7)	416	**0.0069**
*Obesity*						
Sole	52.7 (9.7)	634	0.0767	1.6 (0.9)	369	0.4872
Multiple	57.3 (11.0)	731	0.1771	1.7 (1.2)	411	**0.0411**
*Asthma*						
Sole	55.1 (12.0)	620	0.4330	3.1 (2.1)	366	0.0947
Multiple	61.1 (12.1)	671	**0.0288**	2.3 (1.7)	392	0.7308
*Arthritis*						
Sole^d^	59.8 (8.9)	618	0.3314	2.3 (2.3)	363	0.7391
Multiple	65.6 (8.4)	674	<**0.0001**	1.8 (1.9)	393	**0.0435**
*COPD*						
Sole^d^	60.7 (16.6)	614	0.1614	2.8 (4.2)	359	1
Multiple	64.3 (11.8)	645	<**0.0001**	1.9 (2.5)	376	0.5795
*Thyroid*						
Multiple	66.4 (9.6)	690	<**0.0001**	2.4 (2.7)	402	0.5207
*Neurological*						
Multiple^c^	68.7 (7.1)	611	**0.0006**	1.8 (2.1)	362	0.1463
*Kidney*						
Multiple^c^	69.4 (13.5)	612	0.0706	0.9 (0.6)	364	**0.0064**
*Liver*						
Multiple^c^	56.0 (10.9)	612	0.3878	3.5 (3.5)	361	1

*Chi square analysis with population distribution above or below the mean of the total population (60.1 for age of onset and 2.12 for disease duration). Mean (SD) is in years, represents age of onset or disease duration*.

*Bold represents statistical significance*.

*^a^Control population compared to any antecedent condition population*.

*^b^*N* represents size of population with antecedent condition of interest and control population (no antecedent conditions)*.

*^c^Fisher’s exact test used due to small sample size for age of onset and disease duration*.

*^d^Fisher’s exact test used due to small sample size for disease duration*.

**Table 3:** **The effect of antecedent disease categories and number of antecedent diseases on ALS age of onset and disease duration determined from chi square analysis**.

Antecedent condition	Age of onset			Disease duration		
	Mean (SD)	*N*	*p*-value	Mean (SD)	*N*	*p*-value
**Cardiovascular**						
1	62.8 (11.0)	1014	<**0.0001**	1.8 (1.9)	572	**0.0005**
2	64.9 (11.1)	840	<**0.0001**	1.7 (1.4)	476	**0.0016**
3	63.6 (8.9)	673	<**0.0001**	1.6 (1.7)	386	**0.0091**
4^b^	63.1 (10.3)	614	0.0523	1.9 (1.2)	362	0.4761
Any	63.5 (10.8)	1341	<**0.0001**	1.8 (1.7)	734	<**0.0001**
**Autoimmune**
Sole type	59.8 (11.6)	683	0.1464	2.9 (2.7)	397	0.4189
Multiple types	64.3 (10.8)	849	<**0.0001**	2.2 (2.3)	486	0.0976
**Amount of conditions**						
1	60.9 (11.2)	1007	<**0.0001**	2.1 (2.1)	565	**0.0398**
2	65.0 (10.8)	875	<**0.0001**	1.7 (1.6)	495	**0.0008**
3	66.4 (9.9)	708	<**0.0001**	1.3 (1.4)	413	<**0.0001**
4	63.1 (9.4)	643	**0.002**	2.1 (1.7)	372	0.4483
5^b^	65.6 (6.2)	605	0.0592	3.1 (2.4)	358	1
6^a^^,^^c^	65	601	0.2085	–	–	–

*Chi square analysis with population distribution above or below the mean of the total population (60.1 for age of onset and 2.12 for disease duration). Mean (SD) is in years, represents age of onset or disease duration*.

*Bold represents statistical significance*.

*^a^Sample size of one*.

*^b^Fisher’s exact test used due to small sample size for age of onset and disease duration*.

*^c^Fisher’s exact test used due to small sample size for age of onset*.

#### Age of Onset

Hypertension and hyperlipidemia are found to have a substantially significant older distribution than the control ALS population as the sole antecedent condition and the multiple antecedent condition (*p* < 0.0001). A significant later age of onset distribution is also found for diabetes, asthma, arthritis, COPD, thyroid diseases, and non-ALS neurological diseases in the multiple antecedent condition groups (*p* < 0.05).

In the cardiovascular group, substantial significance for a later age of onset distribution is found for all categories except the four cardiovascular conditions group (*p* < 0.001). Autoimmune conditions only show substantial significance with later age of ALS onset when other antecedent conditions are also present (*p* < 0.0001).

Up to three antecedent condition groups have a substantially significantly older distribution (*p* < 0.0001). Those with four antecedent conditions have a significant older distribution at onset (*p* < 0.05). There is no significance found for those with five or six antecedent conditions, likely owing to small sample size.

#### Disease Duration

Hypertension, hyperlipidemia, diabetes, obesity, arthritis, and kidney disease all have a different distribution than the control for multiple antecedent conditions (*p* < 0.05). The significance found shows that those antecedent condition populations have a shorter disease duration that the control population. Cardiovascular conditions show a difference in disease duration for one, two, and three conditions or having any cardiovascular condition at all (*p* < 0.05). Those categories are correlated to shorter disease duration when compared to the control population. Antecedent conditions of the amounts of one, two, and three result in substantially shorter disease duration than the control (*p* < 0.05). Significance is not found in those with four or five antecedent conditions due to a limiting sample size.

When analysis was repeated with the middle SD omitted for the populations, results mirrored those previously reported. These findings affirm that the potential confound of age, itself, is not substantially skewing the primary results. Thus, the relationships with ALS onset age and disease duration are similar in both the younger antecedent disease ALS patients and the older antecedent disease ALS patients.

### Kaplan–Meier

**Figure 1:**
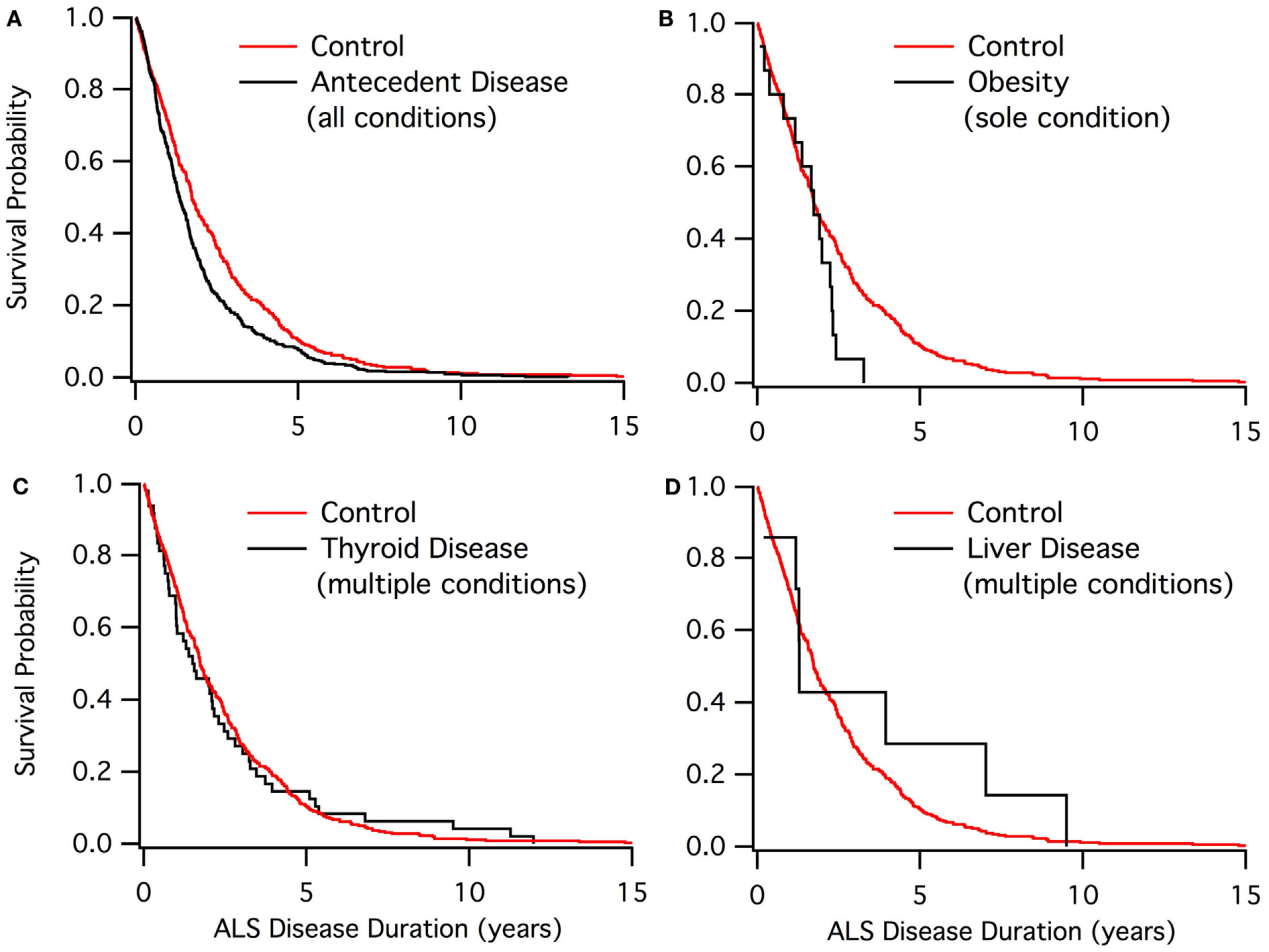
**Kaplan–Meier graphs of ALS disease duration population**. **(A)** Control group (red) compared to those with at least one antecedent condition. **(B)** Control group (red) compared to the obesity sole group. **(C)** Control group (red) compared to those who have thyroid disease. **(D)** Control group (red) compared to those who have liver disease.

Kaplan–Meier graphs resulted in the visualization of several different trends. The overall conditions compared to the control population showed a diversion in the middle of survival, with both groups being near equal at the start and at the tail end of survival. Figure 1 shows different trends throughout multiple conditions.

The Kaplan–Meier results (Figure 1) denoted four main trends for survival. For those with antecedent conditions compared to the control, there is a similar survival rate at the beginning and end of the survival curve between the two groups. The control population exhibited a better survival rate when not at the extremes. This trend was seen in the cardiovascular, blood pressure, and cholesterol groups as well as the antecedent conditions population.

For autoimmune, COPD, and arthritis, there were multiple points throughout the survival curve, in which the antecedent condition group converged with the control group. Nearly every time the curves diverged, antecedent conditions had a lower survival rate.

Non-ALS neurological disease and kidney disease were found to have a much steeper slope (i.e., lower survival) when compared to the control group. The poor results of Kaplan–Meier could be due to either small sample sizes or the antecedent condition already expediting patient death prior to ALS diagnosis.

Asthma and thyroid disease groups were found to have a very similar survival rate and trend compared to the control. Thus, asthma and thyroid disease seem to have no effect on ALS disease duration.

Liver disease was found to have a qualitatively better ALS survival duration than the control. Only the diabetes sole group also included a qualitatively higher survival duration. Nonetheless, neither liver disease nor sole diabetes was found to be statistically significant in extending disease duration, likely owing to sample size.

### Ordinal Logistical Regression

Ordinal logistical regression modeling is used to examine the potential confound of patient age on ALS onset age and disease duration. Hypertension, hyperlipidemia, arthritis, COPD, thyroid disease, non-ALS neurological disease, “other” race, and gender were found to be significant variables with an association to a delayed age of onset past the mean (60.1 years). “African-American” race and obesity are found to have a significant association to an earlier age of onset. When looking at disease duration, age of onset, and “other” race are significant variables associated with shortening disease duration under the mean (2.1 years). See Table 4 for the odds ratios for all variables tested.

**Table 4:** **Ordinal logistic regression modeling of ALS age of onset and disease duration**.

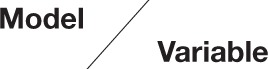	Odds ratio	95% confidence interval	
**Age of onset**			
Hypertension	**0.499**	**0.391**	**0.637**
Hyperlipidemia	**0.416**	**0.316**	**0.547**
Diabetes	0.799	0.526	1.213
Obesity	**1.994**	**1.335**	**2.978**
Asthma	1.283	0.762	2.16
Arthritis	**0.494**	**0.285**	**0.855**
COPD	**0.502**	**0.256**	**0.984**
Thyroid	**0.586**	**0.357**	**0.96**
Neurological	**0.097**	**0.012**	**0.813**
Kidney	0.529	0.151	1.851
Liver	2.795	0.711	10.992
Race (AA)	**1.521**	**1.07**	**2.162**
Race (other)	**0.71**	**0.553**	**0.91**
Gender (female)	**0.64**	**0.508**	**0.805**
**Disease duration**			
Hypertension	1.156	0.847	1.577
Hyperlipidemia	1.106	0.78	1.569
Diabetes	1.18	0.699	1.992
Obesity	1.106	0.647	1.892
Asthma	0.664	0.353	1.251
Arthritis	0.959	0.51	1.801
COPD	1.42	0.601	3.352
Thyroid	0.809	0.454	1.44
Neurological	1.014	0.264	3.893
Kidney	2.451	0.637	9.439
Liver	0.389	0.093	1.629
Race (AA)	0.691	0.441	1.082
Race (other)	**2.016**	**1.479**	**2.748**
Gender (female)	1.186	0.894	1.575
Age	**1.049**	**1.036**	**1.062**

*Parameters include the 11 individual antecedent conditions, race, and gender*.

*Race reference group is Caucasian. Gender reference group is male. For age of onset model, estimates above 1 lower the age of onset. For disease duration, estimates above 1 shorten the duration*.

*Bold represents statistical significance*.

## Discussion

The primary findings of this study support the presence of lower overall antecedent disease in ALS, a later onset of ALS in patients with antecedent disease, and an inverse relationship between onset age and disease duration. We discuss these trends in more detail, including the possible exceptions with liver disease and obesity, as well as potential protective mechanisms. We contend that our findings, combined with current experimental and clinical literature, are highly suggestive of the role of hypervigilant regulation and homeostatic instability in ALS, and conclude with a detailed explanation.

### Antecedent Conditions on Age of ALS Onset

When looking at individual antecedent conditions, every condition of interest is found to have a later age of onset excluding obesity (see What about Obesity?). Hypertension, hyperlipidemia, arthritis, COPD, thyroid disease, and non-ALS neurological disease are found to be significant factors associated with a delay in ALS age of onset. These conditions have also been found to be less prevalent in the ALS population (9, 11, 19).

With the prevalence lower in the ALS population, there may be a mechanism in which these antecedent conditions are protective against ALS in an unknown pathway. Diabetes and hyperlipidemia have been previously suggested to be biochemically neuroprotective against ALS (3, 19), possibly by protecting against the hypothesized ALS hypermetabolic state.

### Antecedent Conditions on ALS Disease Duration

No antecedent conditions are found to be statistically significant factors in predicting the disease duration in patients. Some researchers have found that cardiovascular conditions are beneficial to ALS patients as ALS progresses (16, 20), but further research supports that the conditions do not have a significant impact on the actual ALS disease duration (9, 13, 21). The shorter disease duration found in the antecedent condition groups could be attributed to the fact that those groups have a later age of onset. Advanced age is known to be a predictor of shorter disease duration (2, 4). Another possibility is that hypervigilant regulation (see Hypervigilant Regulation and Homeostatic Instability) exacerbates underlying pathological instability once ALS onset symptoms appear.

Liver disease, while limited by a small sample size, was found to have qualitatively better ALS survival duration than the control. Liver disease’s potential association with increased survival duration, along with its substantially lower prevalence in ALS compared to matched control subjects, is suggestive of the liver playing a systemic, and possibly even a protective role, in the ALS etiology. In fact, a recent study examining ALS treatment with tauroursodeoxycholic acid, a hydrophilic bile acid produced in the liver, showed promise (22).

### What about Obesity?

Antecedent obesity (antecedent static BMI >30) is the only antecedent condition found in the present study to be a significant predictor of a younger age of ALS onset. Interestingly, patients with long-term antecedent obesity prior to ALS onset would, by definition, *not* have hypervigilant regulation (see Hypervigilant Regulation and Homeostatic Instability), which could explain the unique earlier ALS onset age of patients with obesity.

Despite the earlier age of onset, the chi square and Kaplan–Meier analyses show no positive effects on survival in the present study. When other studies have found obesity to be beneficial to survival, the variable measured is usually *rate of change* in BMI (5, 23). Other studies have focused on unhealthy weight, taking any BMI above 24.9 to be considered of interest, with the effects varying as BMI changes (21, 24). Thus, while the rate of BMI change over the course of ALS progression appears to be a predictive measure of disease duration, static assessment of BMI at ALS onset is not. Essentially all research results have agreed that obesity is less prevalent in ALS patients than the general population (11, 21, 24) and, correspondingly, lower BMI is associated with ALS (25).

### Hypervigilant Regulation and Homeostatic Instability

The results of this study supports the contention that the asymptomatic physiological precursors of ALS could actually be protecting against other conditions prior to the onset of ALS symptoms (11), analogous to how one *S* gene infers resistance to malaria without pathological consequence, while two copies result in the pathological sickle cell anemia phenotype (26). Such a contention supports the present study’s primary findings: presence of lower overall antecedent disease in ALS, a later onset of ALS in patients with antecedent disease, and an inverse relationship between onset age and disease duration.

One possible explanatory hypothesis is hypervigilant regulation (11) – the aggressive overreaction of underlying regulatory processes to correct imbalances from homeostasis, making them “hypervigilant” to perturbation (in control theory, a too-high feedback gain). Hypervigilant regulation can quickly compensate to correct small imbalances from homeostasis to initially protect against a wide variety of antecedent conditions (e.g., obesity, hyperlipidemia, etc.) or minimally delay their age of onset. However, hypervigilant regulation increases later susceptibility to more severe pathological homeostatic instability that could directly or indirectly lead to ALS (11). Furthermore, once major pathological instability does occur (e.g., ALS), the overreactive nature of hypervigilant regulation exacerbates further destabilizing regulatory oscillation, which could easily translate into shorter ALS disease or survival durations.

The physically long motoneurons have already been shown to be vulnerable to instability in both physiological and pathological ALS animal models, including the presence of destabilizing somatic dynamics (27), axonal transport deregulation (28), metabolic insufficiency (29), inadequate calcium buffering (29), compensatory oxidative stress responses (29), and dynamic imbalances of pro- and anti-inflammatory cytokines (30). Mathematical oscillations and corresponding instabilities have also been identified in theoretical ALS models (31). The multifactorial nature of ALS, as shown by the variety of underlying cellular and systemic *in vitro* and *in vivo* disturbances in transgenic mice (32) and the presence of overlapping biomarkers with other neurological diseases (6), is also suggestive of system-level instability as a possible primary ALS cause.

## Author Contributions

SH: study design, acquisition of data, completion of statistical analysis, and drafting of the initial manuscript. IO: design of statistical analysis, interpretation of results, and critical revision of the manuscript for important intellectual content. CM: study concept and design, acquisition of data, overall study supervision, interpretation of results, revision of the initial draft, and writing of the final manuscript.

## Conflict of Interest Statement

The authors declare that the research was conducted in the absence of any commercial or financial relationships that could be construed as a potential conflict of interest.
